# Oblique views of chest radiography from a designed rotation angle recommendation increase the contrast ratio between obscured lesions and surrounding structures

**DOI:** 10.1111/1759-7714.13167

**Published:** 2019-08-12

**Authors:** Hui‐Chen Lin, Chung‐Yao Huang, Wei‐Ming Huang, Zong‐Yi Jhou, Chia‐Hung Chen, Yu‐Chan Chien, Chun‐Chao Huang

**Affiliations:** ^1^ Department of Radiology MacKay Memorial Hospital Taipei Taiwan; ^2^ Department of Medicine MacKay Medical College New Taipei City Taiwan; ^3^ Mackay Junior College of Medicine, Nursing, and Management Taipei Taiwan

**Keywords:** Cardiothoracic ratio, chest radiography, contrast ratio, oblique view, pulmonary nodule detection

## Abstract

**Background:**

Chest radiography (CXR) is the main tool used to detect pulmonary nodules. Lateral views of CXR are less effective and the aim of our study was to develop a rotation angle recommendation model to obtain the best oblique CXR with significantly increased contrast between lesions and surrounding normal structures in order to enhance the detection rate for potential obscured lesions on traditional posterior and anterior (PA) CXR.

**Methods:**

A total of 140 subjects receiving low‐dose lung computed tomography (CT) screening were enrolled from the health check‐up database. An additional 14 cases with lung lesions on chest CT were included. Demography was also reviewed. Gross, left and right cardiothoracic ratios (CTR) were measured. All CT images were transformed to CXR to detect the best rotation angles and produce different views of CXR. Contrast ratio was calculated in the transformed CXR from CT with lesions. Comparison of contrast ratio among oblique, posterior‐anterior and lateral views was performed.

**Results:**

CXR shows smaller gross CTR and left CTR but larger heart width and thoracic width in men than in women. Correlation evaluation displays gross CTR, heart width and left CTR are positively correlated with age only for the women group. The most important factor for the best rotation angle is right CTR for left rotation angle and left CTR for right rotation angle. The contrast ratio of the lesion to surrounding structures is significantly better on the oblique views in the designed angles than that on the traditional views.

**Conclusion:**

CXR oblique views in the assigned angle from the 10‐degree rotation angle recommendation are able to enhance contrast ratio between the possible obscured lesions and surrounding structures on CXR.

## Introduction

The prevalence of pulmonary nodules in chest computed tomography (CT) is approximately 17% and the rate increases to 16.8%–73.7% in high‐risk populations.^1^, ^2^, ^3^ Lung cancer is currently one of the most prevalent cancers for both genders and the leading cause of cancer death. Early detection is a major factor which increases the survival rate.^4^ To achieve this goal, posterior and anterior (PA) chest radiography (CXR) and chest CT are the two main modalities in use. Although CT is a powerful modality, CXR has several advantages including a lower radiation dose, it is less expensive, and more widely available.^5^ However, previous studies have reported approximately 18%–32% false negative rate in conventional CXR for the detection of pulmonary nodules.^6^, ^7^, ^8^ The proposed reasons include complex anatomical structures, the effect of overlapping by bone or soft tissue structures, crossings of vessels and ribs, and end‐on vessels.^5^, ^9^

In the past, a lateral view of CXR has been known to provide more information of the retrocardiac, subdiaphragmatic and hilum regions and can be helpful in differentiating suspected lung nodules from nipples or osseous spurs.^10^, ^11^, ^12^ However, the additional diagnostic value for the detection of lung nodules from a lateral view has been proved to be insignificant.^12^, ^13^, ^14^ As a result, chest CT is usually performed after detection of suspected lung lesions on PA CXR.^15^ Although CT is the gold standard in the detection of lung nodules, increased radiation and cost are sometimes of concern.^4^, ^12^ Besides, except for the concept of screening examinations, chest CT scan is preserved for suspicious lesions identified on CXR.

The overlapping effect is a crucial factor diminishing the detection of a lesion because the contrast between lesions and surrounding normal structures is obscured. In this study, we have developed a rotation angle recommendation model to obtain the best oblique CXR with significantly increased contrast between lesions and surrounding normal structures in order to enhance the detection rate for potential obscured lesions on traditional PA CXR.

## Methods

### Subjects

Patients receiving low‐dose lung CT screening were enrolled from the health check‐up database in the MacKay Memorial Hospital, Taipei branch, Taipei, Taiwan between 1 May 2015 and 30 June 2017. The CT scanner used was a Siemens SOMATOM Definition Flash and the parameters for low‐dose CT scan were: Voltage: 120 kVp; Tube current: 80–90 mA; Pitch:1; Rotation time: 0.5 seconds; Slice thickness: 1 mm; Reconstruction interval: 0.7 mm. In order to include images with a wide range of cardiothoracic ratio (CTR), the age range of the included subjects was from 20 to 90 with 20 subjects in each decade and the men and women in each decade were equal, forming a database of 140 subjects. All cases were included continuously but reversely by the study date from 30 June 2017 and the images without complete coverage of the lung or adequate inspiration status were excluded in this study. When there were 20 subjects collected in a specific gender and age decade, no further subject would be enrolled in this specific group in the following subject collection. The final case of the 140 subjects was scanned in May 2015.

An additional 14 cases with identifiable lung‐filled lesions on regular chest CT and CXR were included and the lesions were located in some specific locations where they were at least partially obscured by normal soft tissue structure on CXR, including the bilateral retrocardiac, hilar, subcarinal and subdiaphragmatic regions.

### Measurement on topograms of CT images

**Figure 1:**
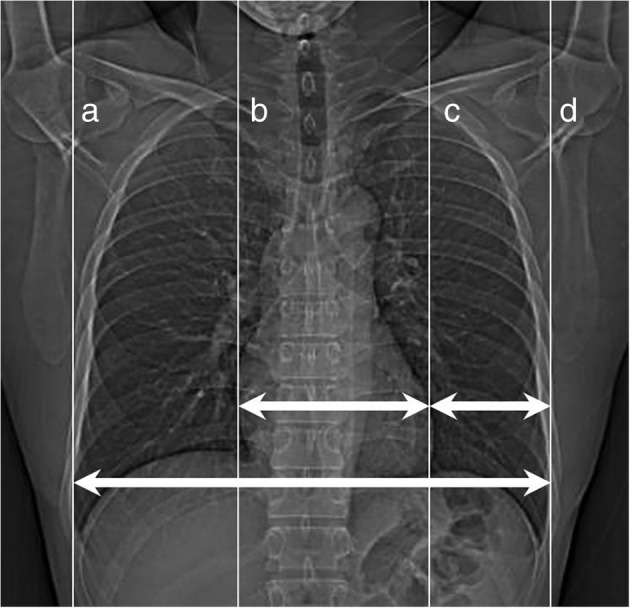
The heart width (distance between [**b**] and [**c**] lines) and thoracic width (distance between [**a**] and [**d**] lines) were measured together with the distance between the left cardiac border ([**c**] line) to the left aeration lung border ([**d**] line). Gross cardiothoracic ratio = **bc**/**ad**. Left cardiothoracic ratio = (**ad**–**cd**–**ad**/2) ÷ (**ad**/2). Right cardiothoracic ratio = (**bc** + **cd**–**ad**/2) ÷ (**ad**/2).

On the topogram of low‐dose lung CT for each 140 subjects, gross CTR was measured by the ratio of the widest cardiac shadow (heart width) and aeration lung field (thoracic width). Right or left CTR was defined as the ratio of ipsilateral cardiac border to the midline of both lungs and ipsilateral lateral margin of aeration lung field to the midline of the both lungs (Fig 1).

### Transformed CXR from CT images

All the CT images were loaded into the integrated tool Pulmo3D_Airways in the Syngo.*Via* post‐processing software environment. By manipulating the contrast, the setting of the first resultant image most visualized like a CXR was saved for the rest of the other CT images.

**Figure 2:**
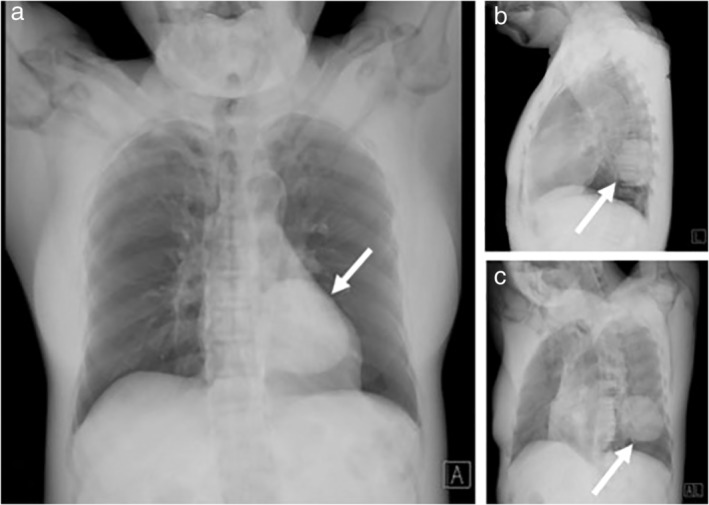
The transformed CXR is demonstrated here using a case with a large left retrocardiac lesion in order to display the appearance of the transformed CXR and the contrast change of the lesion (arrow) in PA view (**a**), lateral view (**b**) and rotated view (**c**).

The transformed CXR was then rotated to the left or right side at increments of 5 degrees. When the ipsilateral cardiac border was touching the ipsilateral side of spinal vertebral bodies, the angles were recorded as left and right rotation angles. The final rotated images were found to have the least overlapped cardiac shadow on the ipsilateral lung parenchyma. The transformed CXR is demonstrated in Fig 2, using a case with a large left retrocardiac lesion, in order to display the appearance of the transformed CXR and the contrast change of the lesion in PA, lateral and rotated views.

### Lesion contrast on different angles of transformed CXR

**Figure 3:**
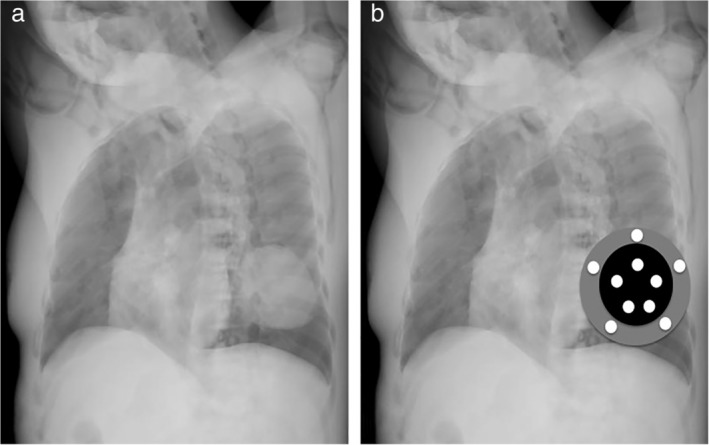
Fig 3(**b**) is identical to Fig. 3(**a**). On Fig. 3(**b**), the black region is the known and identifiable lesion area and the gray region is the perilesional area. The grayscale intensities were retrieved from five points (white spots) in these two areas, respectively. The chosen five white spots are usually distributed in the five points shape of a pentagon. This step was repeated five times with angle change of the pentagon.

The CT images of the additional 14 cases with identifiable lesions were managed using the same method to produce transformed CXR. The PA and lateral views were saved. Based on the analysed results from the previous 140 subjects, the right and left rotation angles were calculated or assigned and then the corresponding transformed CXRs were saved. The calculated angle was directly calculated from the predictive formula and the assigned angle was given based on a designed 10‐degree rotation angle recommendation. All images of PA, lateral, calculated angle and assigned angle views were transformed to grayscale images with 256 different intensities (0: pure black, 255: pure white). Then, for each image, five points were randomly selected from the lesions and perilesional areas, respectively (Fig 3). A chest radiologist with seven‐years experience performed all the selection of region of interest. The contrast ratio was defined as the average of the grayscale intensities of the five lesion points divided by that of the five perilesional points. This procedure was repeated five times and finally there were five contrast ratios for each image.

### Statistical analysis

Statistical analyses were performed using the Statistical Package for Social Sciences program, version 20 (SPSS Inc. Chicago, IL, USA). All parameters were compared between men and women. Correlation between age and the rest parameters was evaluated based on all subjects or men and women subgroups. After controlling gender and age, partial correlation among the parameters was calculated. Stepwise linear regression was used to evaluate the prediction values of the parameters for best left and right rotation angles. Based on the most effective parameters, prediction formula was calculated and then a resultant 10‐degree rotation angle recommendation was designed.

In the additional lesion group, contrast ratio was compared among the images of PA, lateral, calculated angle and assigned angle views using the Games‐Howell test because of significant results on test of homogeneity and the Brown–Forsythe test. A *P* value of less than 0.05 was considered as a significant result.

## Results

### Characteristics of 140 cases

Men and women were equal in subject number. The mean, standard deviation and range of parameters of all subjects were as follows: Age: 54.09 ± 19.42 (20–95); CTR (%): 45.60 ± 7.24 (30.32–64.08); Heart width (cm): 12.06 ± 1.88 (8.2–18.2); Thoracic width (cm): 26.56 ± 2.40 (21.7–33.9); Right CTR (%): 28.45 ± 6.26 (14.19–54.23); Left CTR (%): 62.74 ± 12.57 (33.86–100); Left rotation angle (°): 14.04 ± 6.60 (0–30); Right rotation angle (°): 38.68 ± 8.43 (20–60).

Between men and women, there were significantly smaller gross CTR and left CTR and larger heart width and thoracic width in men (Table 1). Among all cases after controlling gender, there was positive correlation between age and CTR, heart width and left CTR. In the women group, there was positive correlation between age and the same parameters. However, there was no significant correlation between age and the parameters in the men group (Table 2). After controlling gender and age, significant positive correlation was found between gross CTR and heart width (R^2^ = 0.907, *P* < 0.001), between gross CTR and right CTR (R^2^ = 0.519, *P* < 0.001), between gross CTR and left CTR (R^2^ = 0.879, *P* < 0.001), between heart width and thoracic width (R^2^ = 0.175, *P* = 0.040), between heart width and right CTR (R^2^ = 0.475, *P* < 0.001), and between heart width and left CTR (R^2^ = 0.795, *P* < 0.001). In addition, a significant negative correlation was noted between gross CTR and thoracic width (R^2^ = −0.245, *P* = 0.004) and between thoracic width and left CTR (R^2^ = −0.207, *P* = 0.015).

**Table 1:** Comparison of subject heart data between men and women

Group	Men	Women	*P*‐value
Age	54.34 ± 19.31	53.83 ± 19.67	0.876
Gross CTR (%)	44.10 ± 6.97	47.09 ± 7.23	0.014
Heart width (cm)	12.50 ± 2.23	11.61 ± 1.61	0.005
Thoracic width (cm)	28.37 ± 1.69	24.74 ± 1.44	<0.001
Right CTR (%)	28.04 ± 6.82	28.86 ± 5.67	0.440
Left CTR (%)	60.16 ± 10.63	65.32 ± 13.85	0.015

Data are means ± standard deviation.

**Table 2:** Correlation between parameters and age under different classification

	All cases (control gender)	Men	Women
CTR	0.382 (<0.001)	0.150 (0.214)	0.601 (<0.001)
Heart width	0.355 (<0.001)	0.158 (0.191)	0.606 (<0.001)
Thoracic width	−0.027 (0.750)	0.051 (0.673)	−0.118 (0.329)
Right CTR	0.050 (0.556)	−0.030 (0.805)	0.146 (0.228)
Left CTR	0.414 (<0.001)	0.216 (0.072)	0.568 (<0.001)

### Stepwise linear regression result and 10‐degree rotation angle recommendation

All parameters were included to predict left or right rotation angle using stepwise linear regression. For the left rotation angle, there were four parameters with significant predictive value, including the dominant parameter of right CTR with R‐squared change of 0.586. The other three were the left CTR, gender, and thoracic width with R‐squared change of 0.013, 0.015, and 0.016, respectively. For right rotation angle, there were two parameters with significant predictive value, including the dominant parameter of left CTR with R‐squared change of 0.499. The other one was right CTR with R‐squared change of 0.032 (Table 3). Based on the results of stepwise linear regression, the parameter with dominant predictive value was used to calculate linear regression line for each left and right rotation angle. The left rotation angle was calculated from right CTR and right rotation angle calculated from the left CTR. The equations were (1) Left rotation angle = 80.659 * (right CTR) – 8.914, and (2) Right rotation angle = 47.379 * (left CTR) + 8.952. Based on the resultant formula, a 10‐degree rotation angle recommendation was designed. The recommended left rotation angles are: 0 degrees for right CTR 0–11 degrees; 10 degrees for right CTR 12–23 degrees; 20 degrees for right CTR 24–36 degrees; 30 degrees for right CTR 37–48 degrees; and 40 degrees for right CTR more than 48 degrees. The recommended right rotation angles are: 20 degrees for left CTR 0–23 degrees; 30 degrees for left CTR 24–44 degrees; 40 degrees for left CTR 45–66 degrees; 50 degrees for left CTR 67–87 degrees; and 60 degrees for left CTR more than 87 degrees.

**Table 3:** Stepwise linear regression

	Parameter	R	R‐squared	R‐squared change
Left rotation angle	Right CTR	0.766	0.586	0.586
	Left CTR	0.774	0.599	0.013
	Gender	0.784	0.614	0.015
	Thoracic width	0.794	0.631	0.016
Right rotation angle	Left CTR	0.706	0.499	0.499
	Right CTR	0.728	0.53	0.032

### Contrast ratio among different image views

**Figure 4:**
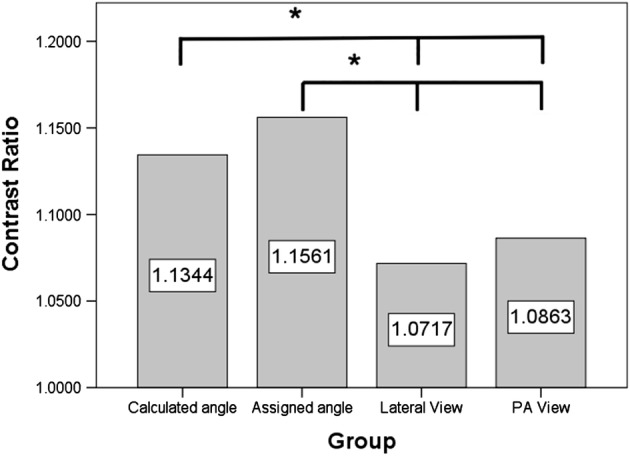
The averages of contrast ratio were 1.1344 in the calculated angle group, 1.1561 in the assigned angle group, 1.0717 in the lateral view group, and 1.0863 in the PA view group. The contrast ratio in the calculated angle group was significantly higher than that in lateral view and PA view groups. The contrast ratio in the assigned angle group was also significantly higher than that in the lateral and PA view groups. There was no significant difference between the calculated and assigned angle groups and between the lateral and PA view groups.

Lesion sites of the included 14 subjects were seven in the left retrocardial region, four in the right retrocardial region, one in the right subdiaphragmatic region, one in the right hilar region and one in the subcarinal region. All lesions were either partly solid or solid tumors with maximum tumor size ranging from 2.7 cm to 8.8 cm. The averages of contrast ratio were 1.1344 in the calculated angle group, 1.1561 in the assigned angle group, 1.0717 in the lateral view group, and 1.0863 in the PA view group. The former two groups had significantly higher contrast ratio than the latter two groups but there was no difference between the former two groups or between the latter two groups (Fig 4).

## Discussion

In comparison with women, CXR of men shows smaller gross CTR and left CTR, but both larger heart and thoracic widths. Correlation evaluation displays gross CTR, heart width and left CTR with positive correlation with age for all cases, or the women group, but not for the men group. After controlling gender and age, gross CTR is positively correlated with heart width, right CTR and left CTR and negatively correlated with thoracic width. Heart width is also positively correlated with thoracic width, right CTR and left CTR. Thoracic width is negatively correlated with left CTR. The most important factor for the best rotation angle is right CTR for left rotation angle and left CTR for right rotation angle. The contrast ratio of the lesion to surrounding structures is significantly better on the oblique views in the calculated or assigned angles than that on the traditional PA or lateral view.

On CXR, our results show that gross CTR and left CTR are both larger in women than in men, but right CTR is similar, suggesting the difference of gross CTR is from left CTR. Furthermore, heart width and thoracic width are both smaller in women than in men. Considering the definition of CTR, the effect of thoracic width is higher than that of heart width in women, attributing to a relatively higher CTR. A previous study shows similar results that women have smaller heart size and thoracic width but higher CTR.^16^ Our results further display the higher CTR is likely due to higher left CTR rather than right CTR.

For all subjects in this study, aging process is positively correlated with gross CTR, heart width and left CTR and these phenomena exist in women, but not in men. The overall finding might suggest that the heart width, especially the left side, significantly increases with age and as a result, left CTR and gross CTR become larger with age in women. Anatomically, the left side cardiac shadow on CXR mainly represents the left ventricle. The prevalence of left ventricular hypertrophy increases with the aging process and the increased rate of the left ventricular size is higher in women than in men.^17^, ^18^ These findings support our results because age‐related enlargement of left ventricular size is faster in women than in men, causing the resultant age‐related increase in heart width, left CTR and gross CTR in women, but not in men. A possible explanation is suggested on the basis of hormone changes in women. Loss of estrogen following the menopause is a risk factor for left ventricular hypertrophy in women because estrogen deficiency is considered to induce inflammation and fibrosis of the heart, resulting in cardiac remodeling and left ventricular diastolic dysfunction and hypertrophy.^19^

Partial correlation analysis after controlling gender and age shows heart width is positively correlated with gross CTR, right CTR and left CTR. Thoracic width is only negatively correlated with gross CTR and left CTR and effect is less than heart width. The results demonstrate that the main effect of gross CTR, right CTR and left CTR is from heart width.

Based on the analytical results, the best rotation angle can mainly be calculated based on right CTR for left rotation angle and on left CTR for right rotation angle. A 10‐degree rotation angle recommendation has been developed based on the estimated formulae for clinical use with ease. In this study, we included 14 cases with known pulmonary lesions located in the commonly obscured location on CXR in order to examine whether the contrast ratio between lesions and surrounding structures was better on oblique views in calculated or assigned angles than on traditional PA or lateral view. Our results show a significantly high contrast ratio on oblique views in calculated or assigned angles than on the traditional PA or lateral view, suggesting that detection is easier on the specially designed oblique views. In addition, there is no significant difference of contrast ratio between the PA and lateral view, probably partially explaining why there is no additional diagnostic value of lateral view on PA view in previous studies. Furthermore, there is no significant statistical difference of contrast ratio between the calculated and assigned angle groups, suggesting a competitive value for these two methods. It is reasonable to apply the simpler method, the 10‐degree rotation angle recommendation, on clinical practices to determine the best rotation angles for patients, by which, the contrast ratio between the possible obscured lesions and surrounding structures on PA CXR can be enhanced and the detection rate can possibly be elevated. However, there is a main limitation for this study. Whether there is significant additional diagnostic value should rely on further largescale studies focusing on the locations prone to obscuration because these locations are the lesion target of this study and obscuration is one of the major factors which causes a diagnostic challenge on CXR.

In conclusion, CXR oblique views in assigned angle from the 10‐degree rotation angle recommendation are able to enhance the contrast ratio between the possible obscured lesions and surrounding structures on CXR and probably elevate the detection rate.

### Disclosure

The authors declare that they have no competing interests.

## References

[1] Gomez‐Saez N , Gonzalez‐Alvarez I , Vilar J *et al* Prevalence and variables associated with solitary pulmonary nodules in a routine clinic‐based population: A cross‐sectional study. Eur Radiol 2014; 24 (9): 2174–82. 10.1007/s00330-014-3249-z. 24962823 PMC4126995

[2] Aberle DR , DeMello S , Berg CD *et al* Results of the two incidence screenings in the National Lung Screening Trial. N Engl J Med 2013; 369 (10): 920–31. 10.1056/NEJMoa1208962. 24004119 PMC4307922

[3] McWilliams A , Tammemagi MC , Mayo JR *et al* Probability of cancer in pulmonary nodules detected on first screening CT. N Engl J Med 2013; 369 (10): 910–9. 10.1056/NEJMoa1214726. 24004118 PMC3951177

[4] Ebner L , Butikofer Y , Ott D *et al* Lung nodule detection by microdose CT versus chest radiography (standard and dual‐energy subtracted). AJR Am J Roentgenol 2015; 204 (4): 727–35. 10.2214/AJR.14.12921. 25794062

[5] Chaya Devi SK , Satya Savithri T . Review: On segmentation of nodules from posterior and anterior chest radiographs. Int J Biomed Imaging 2018; 2018: 9752638 10.1155/2018/9752638. 30498510 PMC6220737

[6] Yerushalmy J . Reliability of chest radiography in the diagnosis of pulmonary lesions. Am J Surg 1955; 89 (1): 231–40. 13218236 10.1016/0002-9610(55)90525-0

[7] Berkson J , Good CA , Carr DT , Bruwer AJ . Identification of "positives" in roentgenographic readings. Am Rev Respir Dis 1960; 81: 660–5. 10.1164/arrd.1960.81.5.660. 13799530

[8] Stitik FP , Tockman MS . Radiographic screening in the early detection of lung cancer. Radiol Clin North Am 1978; 16 (3): 347–66. 746141

[9] Kundel HL . Predictive value and threshold detectability of lung tumors. Radiology 1981; 139 (1): 25–9. 10.1148/radiology.139.1.7208937. 7208937

[10] Lee WS , Yun SH , Chun HK , Lee WY , Yun H . Clinical usefulness of chest radiography in detection of pulmonary metastases after curative resection for colorectal cancer. World J Surg 2007; 31 (7): 1502–6. 10.1007/s00268-007-9060-0. 17483984

[11] Oh JK , Ahn MI , Kim HL , Park SH , Shin E . Retrodiaphragmatic portion of the lung: How deep is the posterior costophrenic sulcus on posteroanterior chest radiography? Clin Radiol 2009; 64 (8): 786–91. 10.1016/j.crad.2009.04.005. 19589417

[12] Kluthke RA , Kickuth R , Bansmann PM *et al* The additional value of the lateral chest radiograph for the detection of small pulmonary nodules‐a ROC analysis. Br J Radiol 2016; 89 (1067): 20160394 10.1259/bjr.20160394. 27605206 PMC5124842

[13] Benden C , Wallis C , Owens CM , Ridout DA , Dinwiddie R . The Chrispin‐Norman score in cystic fibrosis: Doing away with the lateral view. Eur Respir J 2005; 26 (5): 894–7. 10.1183/09031936.05.00059105. 16264052

[14] Collins CD , Padley SP , Greenwell F , Phelan M . The efficacy of a single posteroanterior radiograph in the assessment of metastatic pulmonary melanoma. Br J Radiol 1993; 66 (782): 117–9. 10.1259/0007-1285-66-782-117. 8457822

[15] Osman F , Williams I . Should the lateral chest radiograph be routinely performed? Radiography 2014; 20: 162–6. 10.1016/j.radi.2013.10.006.

[16] Brakohiapa EKK , Botwe BO , Sarkodie BD , Ofori EK , Coleman J . Radiographic determination of cardiomegaly using cardiothoracic ratio and transverse cardiac diameter: Can one size fit all? Part one. Pan Afr Med J 2017; 27: 201 10.11604/pamj.2017.27.201.12017. 28904726 PMC5579422

[17] Cuspidi C , Meani S , Sala C , Valerio C , Negri F , Mancia G . Age related prevalence of severe left ventricular hypertrophy in essential hypertension: Echocardiographic findings from the ETODH study. Blood Press 2012; 21 (3): 139–45. 10.3109/08037051.2012.668662. 22416806

[18] Wild PS , Sinning CR , Roth A *et al* Distribution and categorization of left ventricular measurements in the general population: Results from the population‐based Gutenberg Heart Study. Circ Cardiovasc Imaging 2010; 3 (5): 604–13. 10.1161/CIRCIMAGING.109.911933. 20643817

[19] Li S , Gupte AA . The role of estrogen in cardiac metabolism and diastolic function. Methodist Debakey Cardiovasc J 2017; 13 (1): 4–8. 10.14797/mdcj-13-1-4. 28413575 PMC5385797

